# Supplementary material for: Stoichiometry Dependence of Physical and Electrochemical Properties of the SnOx Film Anodes Deposited by Pulse DC Magnetron Sputtering
Source: Materials (Basel). 2021 Apr 6;14(7):1803. doi: 10.3390/ma14071803 (PMC8038721; doi:10.3390/ma14071803)
Supplement: Supplementary file 1 [file materials-14-01803-s001.pdf]

Support information

Title: Stoichiometry Dependence of Physical and Electrochemical Properties of the SnO_x Film Anodes Deposited by Pulse DC Magnetron Sputtering

Name: Yibo Ma, Xiaofeng Zhang, Weiming Liu, Youxiu Wei, Ziyi Fu, Jiuyong Li, Xuan Zhang, Jingjing Peng and Yue Yan *

S1. Cross–section images and film thickness

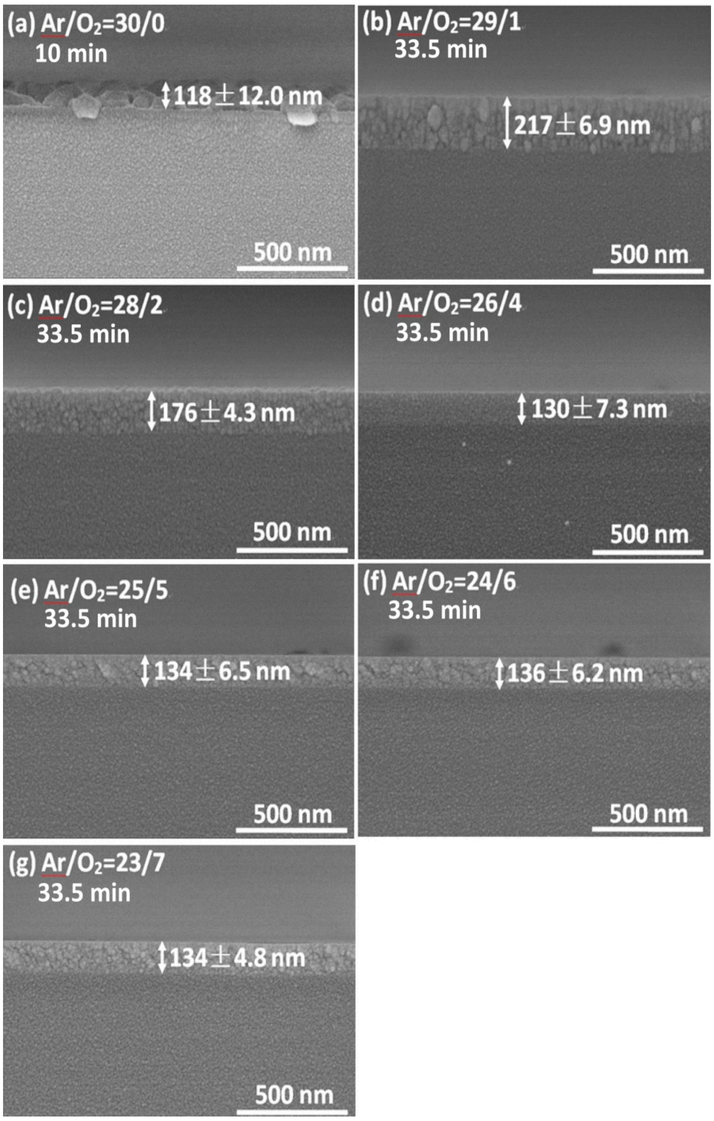

Fig. S1 Cross–section SEM images and film thickness of the SnO_x films deposited at different Ar/O_2 flow ratios: (a) Ar/O_2=30/0, (b) Ar/O_2=29/1, (c) Ar/O_2=28/2, (d) Ar/O_2=26/4, (e) Ar/O_2=25/5, (f) Ar/O_2=24/6, (g) Ar/O_2=23/7

S2. Sn element distribution on the SnO_x film surface before cycling

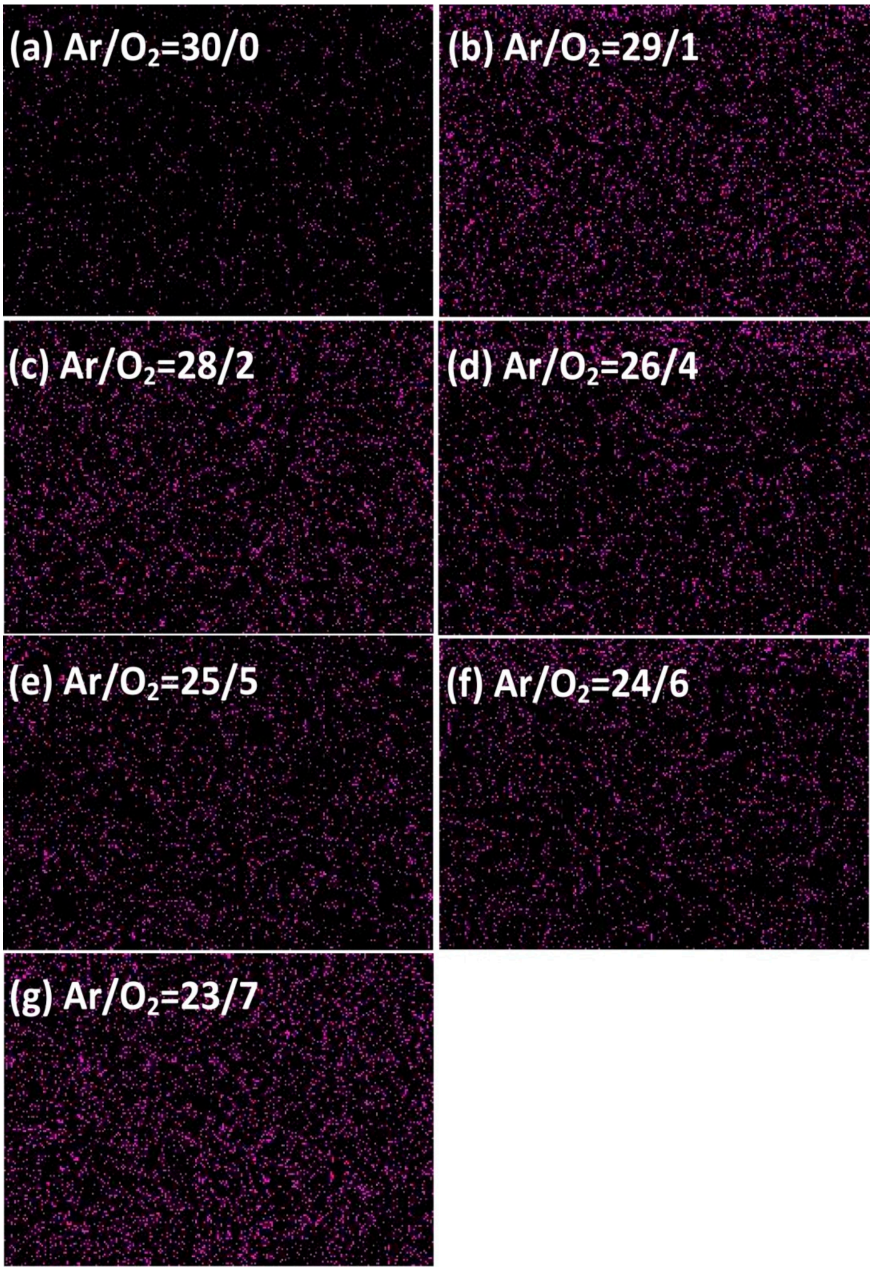

Fig. S2 EDS mapping of the Sn element distribution of the SnO_x films deposited at different Ar/O_2 flow ratios: (a) Ar/O_2=30/0, (b) Ar/O_2=29/1, (c) Ar/O_2=28/2, (d) Ar/O_2=26/4, (e) Ar/O_2=25/5, (f) Ar/O_2=24/6, (g) Ar/O_2=23/7

S3. Sn and O element distribution on the SnO_x film surface after cycling

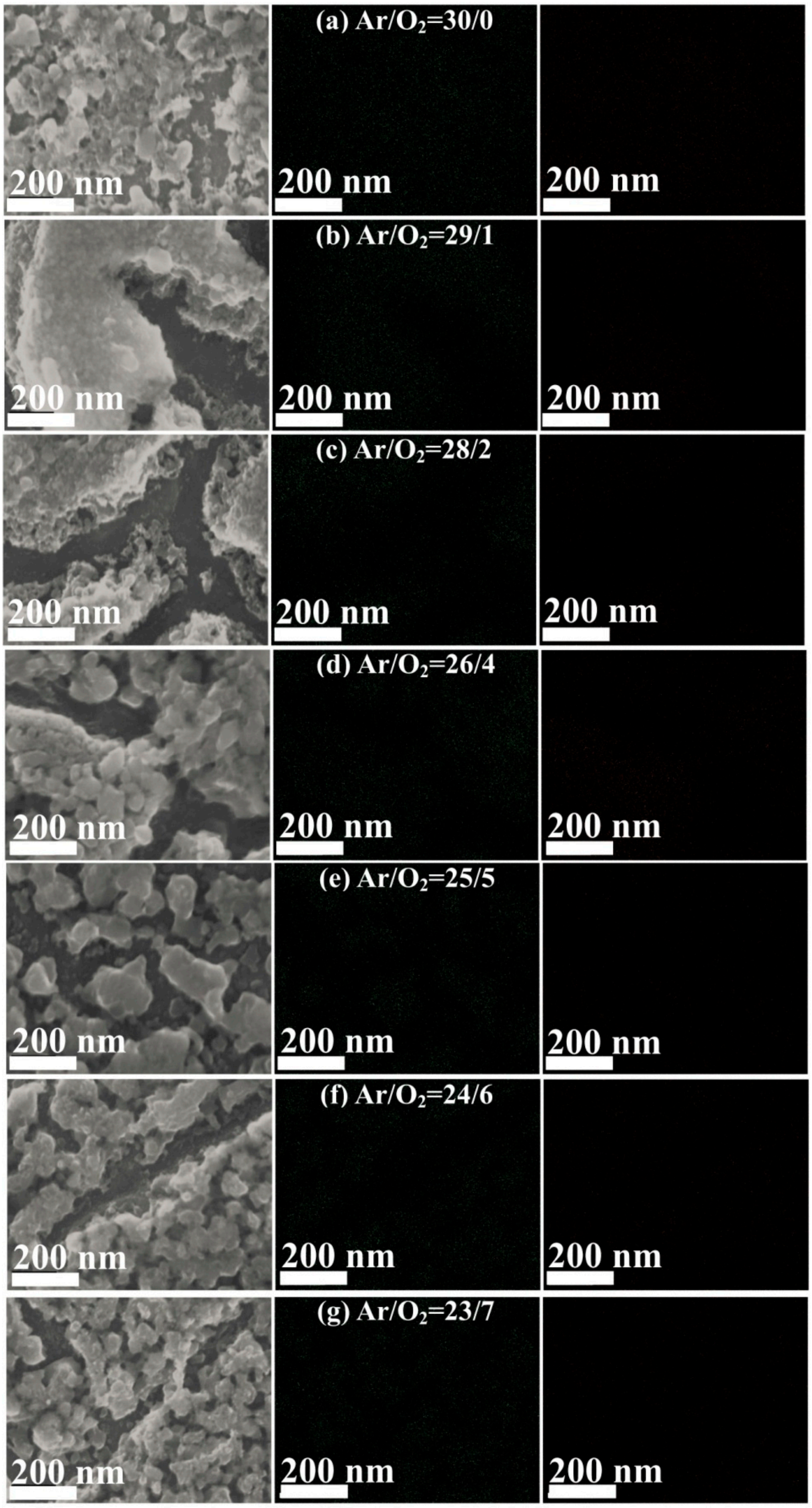

Fig. S3 The local morphology and corresponding EDS element distribution of SnO_x films deposited at Ar/O_2 flow ratio of (a) 30/0, (b) 29/1, (c) 28/2, (d) 26/4, (e) 25/5, (f) 24/6, (g) 23/7 after cycling 20 cycles

S4. AFM images

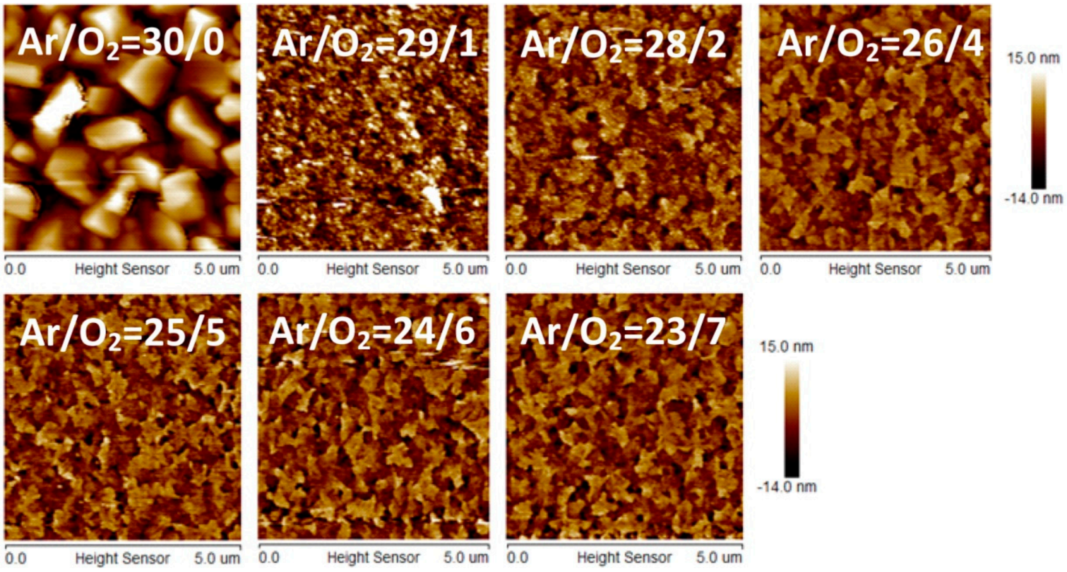

Fig. S4 AFM images of the SnO_x films deposited with different Ar/O_2 flow ratios

S5. XRR measurement principle and its fitting model

The measurement principle is that X–rays are incident obliquely into the film medium at a small incident angle, both refraction and reflection occur on the surface/ interface positions between the film and substrate. Two beams of light reflected on the upper and lower interfaces appear light interference, and periodic oscillation curve of reflection intensity is obtained when changing the incident angle. The fitting procedures are carried out by a complex function containing parameters of film thickness, surface roughness and density [1–3].

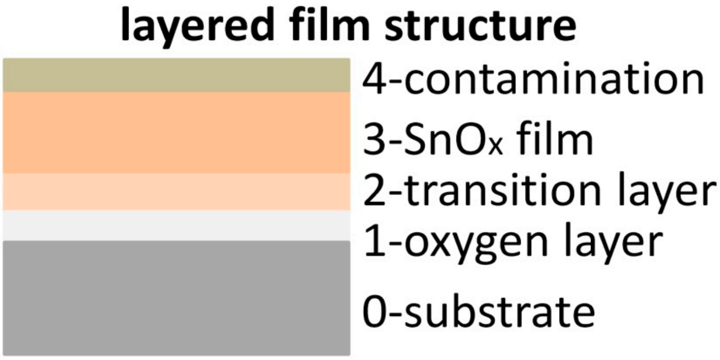

Fig. S5 XRR fitting model: layered film structure

S6. Summary of survey map in XPS

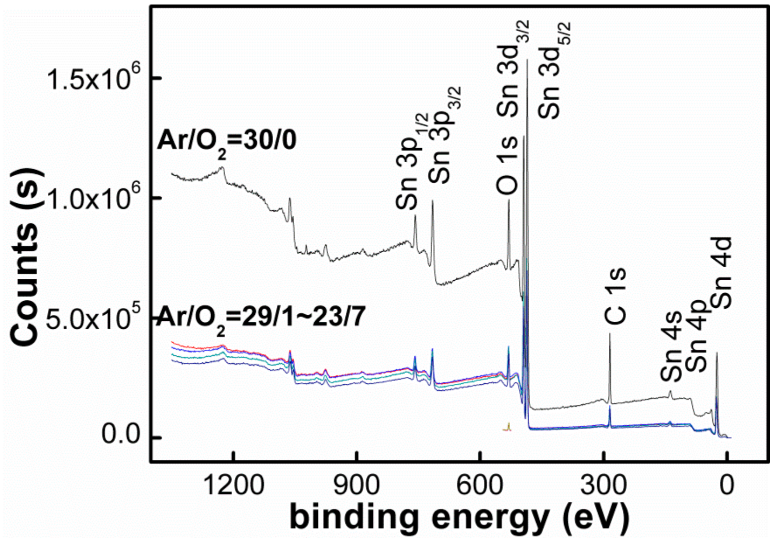

Fig. S6 Survey spectra of the SnO_x films deposited with different Ar/O_2 flow ratios

S7. Comparison of the XPS spectra after Ar^+ etching

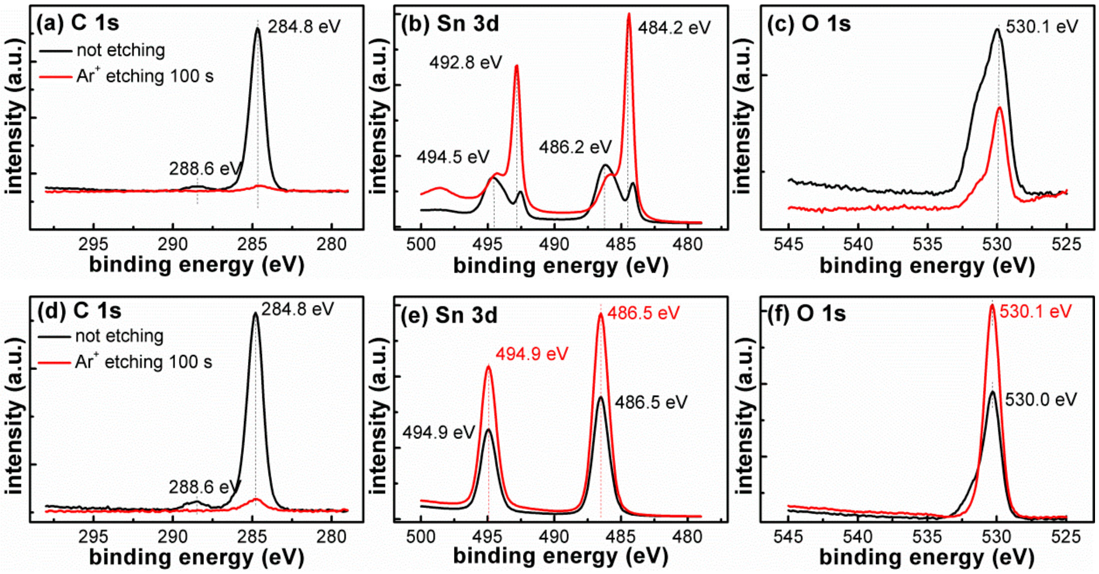

Fig. S7 The XPS spectra of two samples deposited with (a~c) Ar/O_2=30/0 and (d~f) Ar/O_2=23/7, the black line corresponds to the state before Ar^+ etching, and the red line represents the state after Ar^+ etching 100 s.

S8. The XPS spectra of SnO_2 standard material

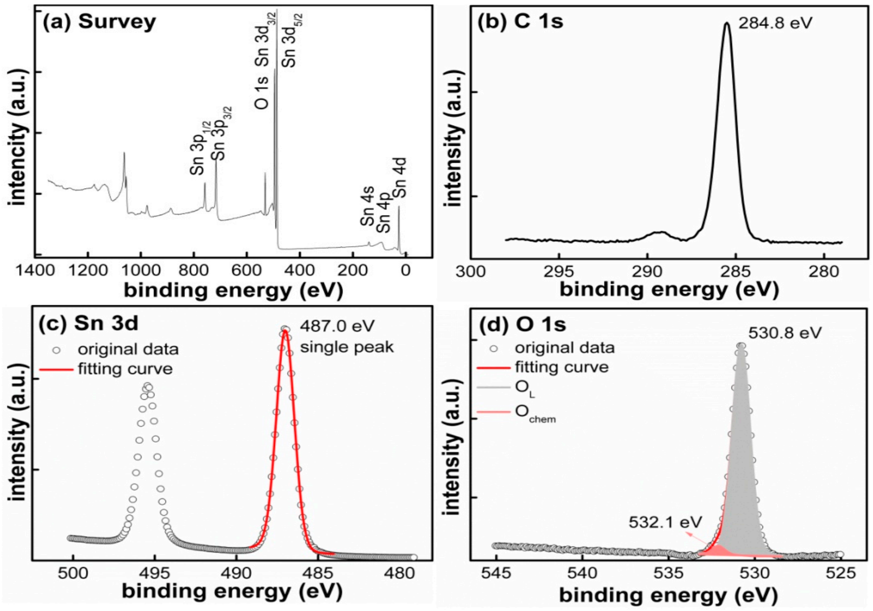

Fig. S8 The XPS spectra of the SnO_2 power standard material.

S9. First charge and discharge curves of the SnO_x film electrodes

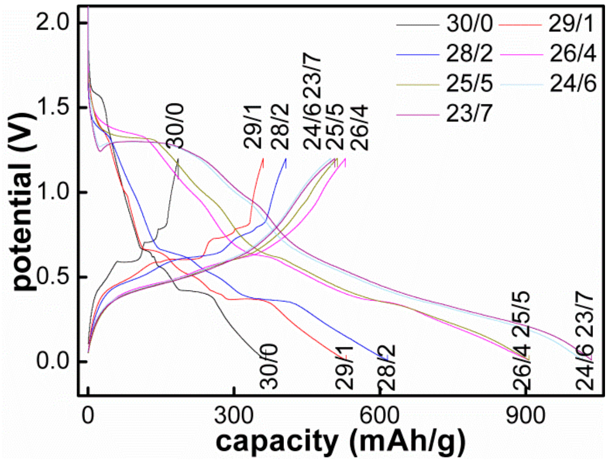

Fig. S9 1st discharge and 1st charge curves of the SnO_x films fabricated with different Ar/O_2 flow ratios, measured at a current density of 44 μA/cm^2, the charge and discharge cut-off voltage range is (0.01~1.2) V

References

[1] Xu, G.L.; Zhao, D.Y.; Liu, G.X. Determining thickness, density and surface roughness of ZnO based film using X-ray reflectometry. PTCA (PART A: PHYS. TEST.) 2010, 46(12), 757−760.

[2] Yu, J.S.; Lu, Q.; Xiao, O.P.; et al. X−ray reflection analysis on the thickness of films. J. Func. Mat. 2008, 39(2), 199−201.

[3] Rauscher, M.; Salditt, S.H. Small−angle X−ray scattering under grazing incidence: The cross section in the distorted−wave born approximation. Phys. Rev. B 1995, 52(23), 16855−16863.
